# Micro- and Nanoplastics in the Environment: Current State of Research, Sources of Origin, Health Risks, and Regulations—A Comprehensive Review

**DOI:** 10.3390/toxics13070564

**Published:** 2025-07-02

**Authors:** Anna Kochanek, Katarzyna Grąz, Halina Potok, Anna Gronba-Chyła, Justyna Kwaśny, Iwona Wiewiórska, Józef Ciuła, Emilia Basta, Jacek Łapiński

**Affiliations:** 1 Faculty of Engineering Sciences, University of Applied Sciences in Nowy Sącz, 33-300 Nowy Sącz, Poland; iwiewiorska@ans-ns.edu.pl (I.W.); jciula@ans-ns.edu.pl (J.C.); ebasta@ans-ns.edu.pl (E.B.); 2 Faculty of Natural and Technical Sciences, John Paul II Catholic University of Lublin, Konstantynów 1 H, 20-708 Lublin, Poland; katarzyna.graz@kul.pl (K.G.); amgronba@kul.pl (A.G.-C.); jacek.lapinski@kul.pl (J.Ł.); 3 Faculty of Medicine and Health Sciences, University of Applied Sciences in Nowy Sącz, 33-300 Nowy Sącz, Poland; hpotok@ans-ns.edu.pl; 4 Department of Environmental Technologies, Cracow University of Technology, 31-155 Cracow, Poland; justyna.kwasny@pk.edu.pl

**Keywords:** plastic waste, polymers, microplastics, nanoplastics, environmental pollution, human health

## Abstract

Small-particle-produced goods, such as those used in industry, medicine, cosmetics, paints, abrasives, and plastic pellets or powders, are the main sources of microplastics. It is also possible to mention tire recycling granules here. Larger components break down in the environment to generate secondary microplastics. Microplastics, or particles smaller than 5 mm, and nanoplastics, or particles smaller than 1 μm, are the products of degradation and, in particular, disintegration processes that occur in nature as a result of several physical, chemical, and biological variables. Polypropylene, polyethylene, polyvinyl chloride (PVC), polystyrene, polyurethane, and polyethylene terephthalate (PET) are among the chemicals included in this contamination in decreasing order of quantity. Micro- and nanoplastics have been detected in the air, water, and soil, confirming their ubiquitous presence in natural environments. Their widespread distribution poses significant threats to human health, including oxidative stress, inflammation, cellular damage, and potential carcinogenic effects. The aim of this article is to review the current literature on the occurrence of micro- and nanoplastics in various environmental compartments and to analyze the associated health consequences. The article also discusses existing legal regulations and highlights the urgent need for intensified research into the toxicological mechanisms of microplastics and the development of more effective strategies for their mitigation.

## 1. Introduction

Microplastics have become ubiquitous in the environment, raising serious concerns among ecotoxicologists due to their potential toxic effects. These particles originate from a variety of sources, including the fragmentation of larger plastic debris (secondary microplastics) and consumer products such as liquid soaps, exfoliants, and cleaning agents. A growing body of evidence also indicates the presence of microplastics in automobile exhausts, primarily as a result of tire and brake wear, which serves as an additional source of atmospheric emissions [1]. The widespread use of plastics, combined with insufficient waste management, poses an increasing threat to the health of ecosystems worldwide [2,3]. Microplastics (MPs) are particles composed of high-molecular-weight polymers that are biochemically stable. Plastics break down into MPs—and even smaller nanoplastics—through various degradation processes, such as exposure to ultraviolet (UV) radiation from sunlight and other environmental stressors [4].

Environmental pollution by microplastics has significant ecological and health implications. These particles can not only be directly toxic to organisms but also serve as carriers of other harmful substances, including pathogens and chemical pollutants. A growing number of studies indicate their ability to accumulate in aquatic organisms, plants, and human tissues, raising legitimate concerns about the health effects of long-term exposure [5]. The purpose of this article is to present the current state of knowledge regarding the presence of microplastics in the environment and to assess their impact on human health.

**Figure 1:**
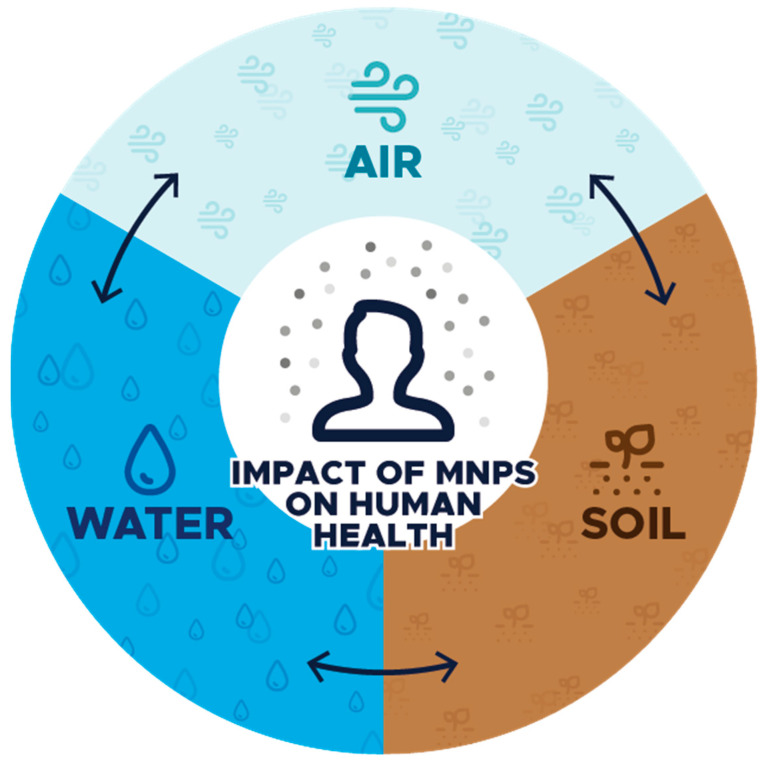
Schematic representation of the impact of microplastics on human health in the context of their environmental presence.

To better understand the potential risks associated with microplastics, Figure 1 illustrates their presence in three major environmental compartments—water, soil, and air—as well as their possible effects on the human body.

Microplastics can enter the human body through three main pathways: water, air, and soil (e.g., indirectly through food or plants). Each of these environmental compartments may serve as a significant source of microplastic exposure, posing potential health risks related to tissue accumulation, toxic effects, or the transport of harmful substances.

## 2. Types of Microplastics (MPs) and Nanoplastics (NPs) and Their Sources

Polymers, due to their diverse physical and chemical properties and wide-ranging applications, are extensively used in both industry and households [6,7,8,9,10]. Ease of manufacture, durability, and low cost are among their main advantages. However, their long decomposition times—such as approximately 450 years for PET bottles and up to 1000 years for other plastic products—are undoubtedly among the most significant disadvantages of plastics [11].

According to Nagalakshmaiah et al. [12] and Leng et al. [13], plastics—or more broadly, plastic materials—are organic polymers derived from oil, natural gas, and coal. In addition to the base polymer, plastics may also contain various modifying additives, such as fillers, heat stabilizers, ultraviolet (UV) stabilizers, flame retardants (e.g., polybrominated biphenyls, PBBs), antistatic agents, foaming agents, and colorants [14].

Among the most commonly used plastics accounting for 90% of total global plastic production (we distinguish polyethylene (PE), polypropylene (PP), polystyrene (PS), polyvinyl chloride (PVC), of which PE is the most common globally and ranks first, PP ranks second and polyethylene terephthalate (PET) is next [13,15]. PET is a plastic used to make plastic beverage bottles, disposable utensils, as well as various types of man-made fibers. Examples of plastics and their sources are presented in Table 1.

## 3. Micro- and Nanoplastic (MNP) Contaminations in the Environment

### 3.1. Presence of Micro- and Nanoplastics in Water

#### 3.1.1. The Scale of Microplastics Pollution in the Water Environment

Water—due to its life-giving and strategic nature—is currently the subject of intensive research regarding the presence of plastic particles and their potential negative impact on the environment and human health. The latest research findings suggest that water pollution by microplastics (MPs) will increase year by year [25,26]. Global plastic emissions from rivers into the oceans amount to between 1.15 and 2.41 million tons annually, with most plastic of the river originating from rapidly developing Asian countries with inefficient waste management systems [27]. Global forecasts show that the concentration of microplastics, which was 2506 mg·m^−3^ in 2016, will rise to 5000 mg·m^−3^ by 2030, and by 2060 it will exceed 10,000 mg·m^−3^ [26].

The threats resulting from the presence of microplastics (MPs) in the aquatic environment most often concern the consumption of microplastics through food and water, which leads to their bioaccumulation in aquatic organisms. There are no clear research results regarding the toxic effects of MP on aquatic organisms. Researcher Ašmonaitė [28] observed a significant decrease in phagocytosis in rainbow trout following exposure to cationic polystyrene (PS) MPs (100–400 μm; 10 mg⋅day^−1^). On the other hand, the findings presented by Bunge et al. [29] suggest that dietary intake of micro- and nanoplastics (MNPs) had a limited impact on fish immune disruption, with no significant changes in immune indicators (head kidney leukocytes) during the exposure periods (9 weeks).

Another potential threat, as in other environments, is the “Trojan Horse” effect, meaning that MP particles adsorb other pollutants present in water and often exhibit synergistic toxic effects once inside the organism. In addition to adsorbing contaminants from the environment, some organic compounds can easily attach to MPs in the aquatic environment within a few hours and subsequently attract microorganisms to form biofilms [30]. Kalčíková G. et al. [31] found that MPs attached to biofilm (180.5 ± 118.7 μm) can adsorb almost 1.5 times more silver within seven days compared to clean MPs. Another study showed that MPs can become vectors for pathogen transmission. Kesy K. et al. [32] demonstrated that polypropylene (PP) carries Gram-negative *Vibrio* bacteria, which cause coral disease.

Increased plastic production and its slow natural degradation have led to its accumulation in the aquatic environment—both in freshwater and marine ecosystems. As a result, micro- and nanoplastics negatively impact aquatic organisms [3]. The amount of plastic waste deposited in the sea is increasing year by year, which leads to their increased aggregation on the seabed and in the water column [33,34,35,36,37,38]. Plastic deposited in the oceans can persist for hundreds or even thousands of years, undergoing mechanical, chemical, biological, and photochemical degradation processes that lead to the formation of microplastics (MPs) (<5 mm) and/or nanoplastics (NPs) (<1 μm) [15,39].

PET is a plastic commonly used in the production of bottles, synthetic fibers, and disposable tableware. Their density (1.37–1.45 g/cm^3^) causes them to quickly sink to the bottom, becoming accessible to benthic organisms [40]. In the marine environment, PET undergoes fragmentation and likely abiotically weathers through photooxidation and hydrolysis [33]. A very important factor potentially influencing the rate at which chemical compounds leach from PET (and thus its potential toxicity) is the fluctuation of pH levels in the marine environment [41].

#### 3.1.2. Impact of Micro- and Nanoplastics on Aquatic Organisms and Human Health

Due to their resemblance to certain types of zooplankton and food particles, micro- and nanoplastics (MNPs) are often ingested by fish, entering their digestive systems. Once inside the organism, they do not remain confined to the intestines; instead, they infiltrate the fish’s circulatory and lymphatic systems, eventually spreading throughout various tissues and organs. Microplastics have been found in the gills, muscles, liver, heart, swim bladders, ovaries, spinal cords, and even the brains of fish. The presence of MPs in these organs is associated with significant negative effects, including disorders of the reproductive, neurological, hormonal, and immune systems. Aquatic organisms develop various diseases either directly due to MPs or as a result of their interactions with other pollutants [4].

Yang P. et al. [42] studied the accumulation capacity of microplastic particles by freshwater copepods (originating from Lake Baikal in Siberia) both in natural conditions and in the laboratory. Copepods dominate zooplankton biomass in large lakes, where they hold a critical trophic position, channeling energy from planktonic and microbial food webs to higher trophic levels. The concentration of MPs in Lake Baikal is high compared to other large lakes. The occurrence rate of MPs was over 10 times higher than that recorded for copepods in lakes in British Columbia and comparable to the frequency observed in oceanic areas recognized as microplastic pollution hotspots. The high occurrence rate may be attributed to the detection of smaller, more numerous MPs by the researchers [42], which were often not detected in other studies.

All MPs ingested by copepods were fibers or fragments, with an average particle size of 65.2 ± 41.9 μm; transparent MPs were the most common; and the most frequently ingested MPs consisted of high-density polyethylene terephthalate (PET). The researchers [42] emphasize that calanoid copepods may be potential vectors for transferring MPs into the digestive systems of organisms in large, oligotrophic lakes.

Due to their properties and structure, microplastics and nanoplastics can serve as carriers of microbiological contaminants for aquatic organisms [43,44]. MPs can also accumulate, release, and increase the concentration in water of chemical compounds such as dichlorodiphenyltrichloroethane (DDT), polybrominated diphenyl ethers, and other substances [45,46,47,48,49].

The size of nanoplastics allows them to be absorbed by fish and other aquatic organisms, potentially disrupting their basic physiological functions. Marine organisms (including fish) in turn move through the food chain and may pose a threat to human health [50]. Cozar et al. investigated the uptake of microplastics by marine organisms [51]. The researchers concluded that there is insufficient evidence of microplastic bioaccumulation in marine organisms, as they are rapidly excreted by many marine species. However, Marn et al. found that the presence of microplastics in seawater had negative effects on a group of 700 aquatic species worldwide (including penguins, crustaceans, and sea turtles), resulting in reduced food intake, developmental disorders, and behavioral changes [52]. Pabortsava and Lampitt noted that the impact of microplastics on organisms living in remote areas of the oceans has still not been sufficiently studied [53].

Udayakumar et al. found that microplastics without modifying additives are not potential carriers of chemical compounds or microorganisms and are not chemically hazardous to aquatic organisms, but they do cause issues related to physical discomfort, such as intestinal blockage [54].

In the production of some types of nano- and microplastics, specific chemical additives are used that affect the ability to adsorb pollutants from water to the surface of microplastics, thanks to which they can mimic carriers capable of penetrating the cell membranes of aquatic organisms. Numerous studies reveal the harmful effects of micro- and nanoplastics on aquatic animals and their health [33,55,56,57].

Lee and Kim, in their research, demonstrated the negative impact of microplastics on freshwater organisms such as Pseudobagrus fulvidraco, which inhabit lakes and swamps. The observed effects included intestinal, gill, and liver problems [58]. Zhang et al. [59] reported the adverse effect of MPs in inducing oxidative stress in Ictalurus punctatus, while Yedier et al. [60] observed changes in antioxidant levels in Cyprinus carpio under the influence of MPs. Among aquatic invertebrates, chronic exposure to MPs affected the growth, reproduction, and mortality of Daphnia magna [61]. Decreased fertilization rates and a high degree of larval dysfunction were also observed in Mytilus galloprovincialis due to prolonged exposure to MPs [62], and the interaction between CO_2_, temperature, and MPs increased the mortality of Phylloicus [63].

Bioaccumulation of MPs is a highly debated topic, as it may indirectly affect humans through the consumption of aquatic organisms such as fish [64]. Contamination by heavy metals and microplastics is common in freshwater ecosystems. Recent studies have shown high concentrations of Cd, Pb, Zn, Cr, and Cu associated with microplastics, which act as vectors of pollutants, including heavy metals, due to their adsorption capacity [65,66], thereby increasing their potential toxicity to aquatic ecosystems.

A summary of the polymers detected in the aquatic environment and their sizes is presented in Table 2.

#### 3.1.3. Migration Pathways of Microplastics into the Aquatic Environment

Studies have shown that less than 80% of plastic waste found in the aquatic environment comes from land-based sources, with the largest shares coming from households, industry and coastal activities [67].

The entry of micro- and nanoplastics (MNPs) into the aquatic environment, including marine ecosystems and freshwater surface waters [68,69], is mainly caused by human negligence (littering), uncontrolled discharges of domestic and industrial wastewater from treatment plants, runoff [35,70,71,72,73,74], and ineffective plastic waste storage at landfills [75].

According to Park H. and Park B.S., many countries around the world attempt to treat wastewater using conventional biological and chemical treatment methods; however, these methods are not optimized for removing micro- and nanoplastics, making wastewater a significant source of such pollutants [67]. Another major issue is the management of sewage sludge from treatment plants, as the processes used to stabilize the sludge do not affect the physicochemical properties of the tested microplastics. Sludge containing microplastics may end up in landfills, from which they can be washed out by, for example, rainfall and subsequently enter the environment [76].

### 3.2. Presence of Micro- and Nanoplastics in Soil

#### 3.2.1. The Scale of Microplastic Pollution in the Soil Environment

There are two approaches to the topic in microplastic research. One focuses on current effects; the other perspective focuses on possible future consequences. Microplastics are an emerging global agent of change that impacts soil properties, plant productivity and ecosystem processes. Its presence in the environment is related to human activity and can be treated at the same level of significance as increased levels of carbon dioxide in the atmosphere.

The occurrence of microplastics in soil ecosystems remains largely unexplored. Microplastics enter the soil through various routes, including agricultural activities such as the use of sewage sludge, compost [77,78,79,80,81], urea fertilizers and sewage used for irrigation [81,82]. Other anthropogenic factors that cause soil contamination with microplastics include road traffic and sewage containing vehicle tire particles, as well as waste disposal in poorly designed landfills, i.e., without a drainage system. Microplastics can also enter the soil via atmospheric air movement, as they exhibit high aeolian mobility. Agricultural soils can act as a temporary sink or a dynamic source of wind-borne microplastics through wind-induced erosion [82]. The wind-blown street dust is also significant, as it helps to spread the pollutants to further areas. Biodegradation of microplastics in soil usually occurs through the colonization of the plastic surface by microorganisms and depolymerization into mono- and oligomers through enzymatic hydrolysis [83,84].

**Figure 2:**
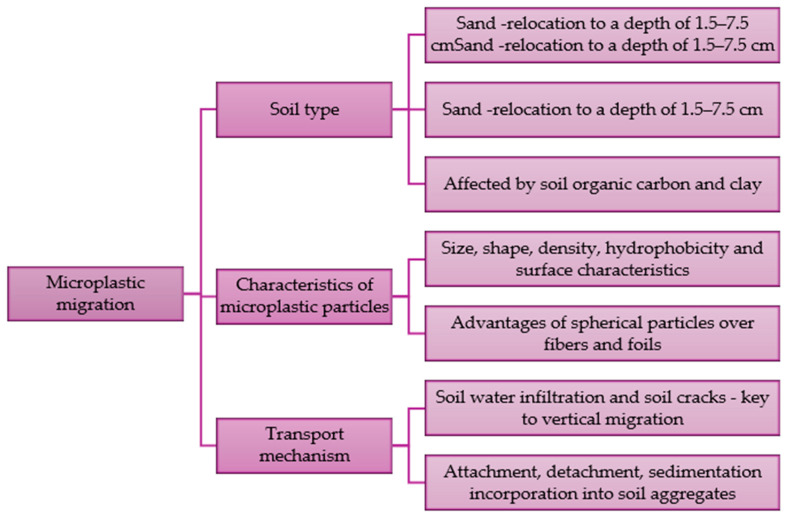
Factors influencing the migration of microplastic particles [82].

The migration of microplastics in soil can occur both vertically and horizontally and is regulated by various factors. If the size of microplastic particles is larger than the soil pore diameter, they will be retained in the area. It has been shown that high-density plastic particles and small particles can easily pass through the pores. They also reach deeper layers of soil. In column tests, particles with an average size of about 21 μm migrated to a maximum depth of about 7.5 cm. These particles reached a greater depth than particles with a size of about 349 μm [85]. The migration and retention of microplastics are influenced by soil properties. Organic matter content and soil structure are found to be important. Sandy soils tend to allow for greater microplastic movement compared to clay-rich soils because they have less cohesion. The transport of microplastics in the substrate, regardless of what it is made of, depends on the roughness of the medium surface, the presence of specific microorganisms (biofouling), organic matter, saturation and hydrodynamic conditions. Increasing ionic strength can accelerate migration [82]. Factors influencing the migration of microplastics in soil are shown in Figure 2.

The type of substrate and its infiltration capacity, the physicochemical properties of microplastic particles and the transport mechanism are key factors determining the migration of these contaminants. The spherical shape of microplastics accelerates the vertical migration of particles into deeper soil layers. However, there are no known studies explaining the effect of shape on the movement of microplastics in the soil.

The influence of the shape and type of microplastics on their moving and retention in soil requires further research. The migration of microplastics may be limited by various mechanisms, depending on their properties, as well as environmental factors, which will cause various interactions to occur. The phenomenon of sorption at liquid-solid surfaces as well as at the solid-air interface, various types of collisions of microplastic particles with soil, biofilm deformation and pore filling are considered to be the key processes explaining the movement and retention of microplastics [82].

#### 3.2.2. Properties of Microplastics in Soil and Their Impact on Soil Processes

##### Physicochemical Properties of Microplastics and Their Interactions with Soil

The accumulation of microplastics in the soil changes its physicochemical properties, affecting its structure and fertility, and consequently reducing its quality [82,86,87,88]. Microplastics are characterized by a surface charge, large surface area and hydrophobic properties, therefore they have an electrostatic repellent effect on soil components, i.e., organic matter and clay, and thus can easily migrate in the soil [88,89,90,91]. As a result of the migration of microplastics in soil, they can enter groundwater [92]. Microplastics can adsorb various types of inorganic and organic pollutants, e.g., pesticides, pharmaceutical products and heavy metals [89,93,94,95]. The adsorption mechanism is influenced by factors such as the type, structure and size of microplastics, the properties of adsorbates and environmental conditions, i.e., pH and salinity [89,96]. The adsorption of these substances onto microplastics is dependent on pressure and temperature. High pressure increases the solubility of pollutants, while high temperatures increase the adsorption kinetics [89]. Due to their high stability, small size and high mobility, microplastics can act as carriers of other pollutants, e.g., heavy metals, and contribute to their accumulation in soil [89,97]. Microplastic surfaces adsorb titanium and iron oxides and clay minerals from soil [88]. Leaching of additives such as BPS, BPA, phthalates used in the production process of plastics as a result of factors such as UV radiation, negatively affects soil flora and fauna [82]. The mechanisms responsible for the impact of microplastics on diversity of microorganisms and greenhouse gas emissions are complex and not well understood.

The accumulation of microplastics in soil is a factor influencing the content of organic matter, namely organic carbon. The formation, stability and decomposition of soluble organic carbon in soil depend on the presence of microplastics [98]. Microplastics also affect ion (cation) exchange processes occurring in the soil and the activity of groups of microorganisms, thus affecting soil chemistry. To better understand the influence of organic matter on microplastics, an analysis focusing on the content of organic matter and microplastic carbon in soil was conducted. Microplastic carbon concentration (MPC) is calculated in two steps. First, the qualitative concentration of the individual plastics must be calculated, and then the MPC is calculated based on the proportions of carbon atoms in the different types of microplastics, taking into account the particle shape [98,99,100]. Microplastic carbon is more resistant to microbial degradation than organic matter (e.g., plant residues), despite the fact that microplastic carbon constitutes only a small part of the total organic carbon in soil. This is due to the high carbon content (80%) in its molecular structure. In the presence of increased microplastic carbon content, there is a relatively low level of soluble organic matter decomposition, limited formation of autochthonous products, and strong humification. Microplastics have been shown to potentially reduce dissolved organic carbon in soils [98].

##### Influence of Microplastics on Soil Fertility and Structure

Microplastics also affect other parameters that determine soil fertility, such as pH, and the content of nitrogen and phosphorus in forms available to plants. The addition of microparticles of PE, PS, PA, PLA, PBS, PHB to the soil at concentrations of 0.2 and 2% caused changes in the content of nutrients: increase in soil pH with 2% PLA and PHB dose and decreased by 2% PE and PS; increase DOC content at low dose of PE, PS, PA and PBS; decrease in nitrate nitrogen content; no change in P concentration at 2% PE and PS, and 0.2% PHB, but decrease at the remaining doses [101].

According to literature sources [88,89,102,103], not only the presence of microplastics affects soil properties, but also the shape of particles. Contamination in the form of fibers can cause disturbances in soil structure, which affects its porosity and water permeability. As a consequence, this contributes to the limitation of air flow in the soil, which negatively affects the processes related to microbiological activity [83,89,103]. The increased hydraulic conductivity is a consequence of adding microfibers to organic matter, which increases macropore stability [104]. Microplastics such as PP, PET, PES, PMMA and HDPE, when present in clay-sandy soil, will cause a decrease in bulk density in the soil outside the rhizosphere, while an increase was observed in the rhizosphere soil [82]. The presence of PET microparticles in soil causes changes in soil properties, i.e., lower pH, reduced soil organic carbon content and reduced nitrogen availability. High concentrations of PET microplastics in soil affect soil permeability and water retention capacity, which results from disruptions in soil aggregation. [89,105]. This effect is correlated with the concentration of microplastics; the more microplastics, or more precisely polyester microfibers, are present in the soil, the more the bulk density decreases [82]. Microplastics in the form of films can reduce soil aggregation, which leads to a decrease in soil structural stability. In surface soils, the content of microplastics increases significantly with decreasing aggregate stability in different land use categories. The weaker corrosion resistance is a consequence of the low stability of soil aggregate [82]. The presence of PE films in the soil causes a decrease in water retention, which consequently leads to a reduction in water availability during the intensive growth phase of plants [89,106]. It has been shown that the shape of MP can affect the chemical and physical structure of the soil (mainly foils) and also act directly on plants, e.g., by causing mechanical damage to roots or directly affecting microorganisms, as is the case with fibrous MPs [82].

##### Microplastics in Coastal Ecosystems and Vegetation-Based Capture

The presence of microplastics has been confirmed [98] on coral reef islands of the Chinese Xisha Archipelago in the South China Sea. These islands are in different stages of development and experience different levels of human activity, making them an ideal place to study the environmental characteristics of microplastics. The abundance of microplastics ranged from 1068 to 1616 particles/kg in different reef islands. Other literature data reported 610 pieces/kg and 682 pieces/kg in sand and sediment of Xisha Islands, respectively [107,108]. The main types of microplastic polymers were PA, PP and PE. The dominant types of microplastic polymers in the vegetated site were PE (5.96%), ACR (acrylic polymer) (4.32%) and PA (3.03%). The main types of polymers in the poor vegetation site were PP, PVC, EVA and PE with contents of 29.8%, 19.78%, 13.09% and 6.72%, respectively. It was found that the content of MP increased in the study areas as the island developed. The conditions on tropical reef islands, i.e., high temperature, high solar radiation and high salinity, lack of vegetation, in the sampling locations contribute to the acceleration of the aging process of plastics. Plastic waste can also be degraded by natural factors, such as strong winds and heavy rains, as well as by some organisms, such as crustaceans and birds living on the islands. These factors may contribute to the finer nature of microplastic particles in Xisha soils, as confirmed by the cited [98] studies. Higher proportions of finer microplastic fractions potentially pose a greater threat to the delicate ecosystem of the reef island, as they are potentially harmful to the soil ecosystems of the islands, and also have a higher potential for migration into the marine ecosystems of the coral reefs, further posing a higher risk to marine life over a wider area [98]. The location of microplastics on islands was significantly associated with human activity. A study of microplastic content in different locations on the atoll revealed that ocean currents and monsoons are the main drivers of microplastic accumulation on coral reef islands [98].

In field studies of microplastic contamination of a given area, the so-called Environmental Index (EI) is used [109]. EI determines the degree of microplastic enrichment in a place covered with vegetation in relation to a place without vegetation and is calculated using the following Formula (1):

(1) $$EI=\frac{A_{V}}{A_{b}},$$

where

$A_{V}$—microplastic content in a place covered with vegetation,

$A_{b}$—microplastic content in a place without vegetation.

Based on the EI value, it can be concluded that a sampling site covered with vegetation is more likely to have an index above 1. If EI > 1, it means that microplastics were retained in the territory and trapped by the vegetation there. Table 3 presents the EI values determined for selected plant species, enabling the assessment of their sensitivity to the presence of microplastics in the soil environment.

The higher accumulation of microplastics in seagrass beds and mangrove forests compared to the area without vegetation is in line with expectations. This is due to higher sedimentation of particles, probably caused by longer travel paths, increased friction and energy dissipation, which causes, for example, faster settling during airborne transport. Cesarini and Scalici [110] studied the accumulation of microplastics in river riparian areas in inland habitats in central Italy. In total, the authors found 483 plastic items in the vegetated area and only 51 plastic items in the non-vegetated area. The EI value in this case was as high as 17.6, which resulted from the greater ability of lush bushes to capture microplastics compared to herbaceous plants. Additionally, the authors showed that the presence of organic matter in the soil also affects the capture efficiency and accumulation of microplastics in the soil. The uptake of microplastics in inland ecosystems mainly relies on trees and shrubs, whereas mangroves capture microplastics mainly through tree canopies, aerial roots of plants, and sources of particulate organic carbon such as drifting macroalgae, terrestrial organic matter including plant and wood debris [98,110]. It can be seen that the EI in this study of coral island areas is relatively low because shrub vegetation on the island is not abundant and the study area is located at a considerable distance from facilities related to human activity. In addition, there is a relatively low level of soil development and the soil substrate consists mainly of coral sands with a large particle size and a relatively low content of soil organic matter; therefore, the capacity of the surface soil to accumulate microplastics on coral reef islands is lower than in other ecosystems. Mangrove vegetation and seagrass beds can reduce the impact of waves and currents on coastal areas and will also help to limit the spread of MP further inland [98].

Knowledge of the relationship between the abundance and type of vegetation and the ability to capture microplastics can be used in spatial planning and development of areas around point and area sources of plastic pollution. One of the main sources of microplastics in soil is sewage sludge used for fertilization purposes.

##### Wastewater Sludge as a Source of Microplastics in Agricultural Soils

The use of sewage sludge for fertilization increases the accumulation and mobility of microplastics in the soil [82,88]. Sewage sludge is often used for fertilizing agricultural land (27.1%), land reclamation (3.4%) and as input to composting plants (3.9%) [89]. Microplastics contained in sewage sludge are released into the soil environment and therefore the sludge becomes an important source of microplastic pollution in soil [89]. According to statistics reported by Zhou et al. [88], about 3.12 million tons of dry sludge are used as soil amendment in China every year, and at least 1.7 × 10^13^ microplastic particles are injected into the soil annually. In Europe, the use of sediment for fertilization purposes is estimated to result in soil microplastic contamination of 8.6–71 × 10^13^ microplastic particles/year [88,111]. Zhou et al. [88] showed that the microplastic abundance in the sediment used in their study was 8660 particles/kg. This value was within the range of 1500–11.000 particles/kg of sewage sludge for several Chinese cities, with an average of about 5500 particles/kg of sludge. In Europe, the average microplastic count in sewage sludge was about 24.700 particles/kg [111]. The number of microplastics in sewage sludge was, for example, 6.08 MP particles/g dry weight of sludge in Norway, 4.02–15.4 MP particles/g dry weight of sludge in Ireland [112], 13.8–116.3 MP particles/g dry weight of sludge in Poland [89], 16.7 ± 1.96 MP particles/g dry weight of sludge in Sweden [112], and for Germany, Spain and Italy: 1–24; 133 ± 59; 133 MP particles/g dry weight of sludge, respectively [113,114,115]. In Scotland, microplastic content ranged from 14.035 ± 1.149 particles/kg to 41.880 ± 2.669 particles/kg of dry sludge [116]. Microfibers accounted for 65% of all plastic forms in sewage sludge [116].

The presence of microplastics in sewage sludge and differences in its abundance are influenced by factors such as population density, processes occurring in sewage treatment plants, the presence of industrial plants producing plastics, seasonality, plastic consumption, as well as sampling and analysis methodology [89,117]. The most common types of microplastics in sewage sludge are PP, PE, viscose, polyester, nylon and polyethylene terephthalate (PET). The highest percentage of sludge samples was viscose 31%, followed by polyester 23% and PE 15%. The percentage of PET, PP and nylon was 8% and the percentage of PE:PP was 7%. This microplastic composition in the sludge samples was mainly due to the large amount of viscose and polyester components in the clothes, which were sent to the sewage treatment plant along with the wastewater from the domestic laundry, causing them to remain in the sludges [118].

Zhou W. et al. [88] studied the effect of sewage sludge fertilization on the content of microplastics in soil. In a separate experimental field, the effect of the amount of sewage sludge applied on the accumulation and migration of microplastics in two soil layers was evaluated, the upper layer was from 0 to 20 cm in depth, and the lower layer was from 20 to 40 cm. After the sludge application of 36 t/h per year for 16 years continuously, the microplastic content and migration coefficient in the topsoil layer reached 6811 particles/kg and 148%, which were approximately 5 and 20 times higher, respectively, than in the control soil without sludge. Microplastics in the upper and lower soil layers were mainly 0.2–0.5 mm in size, mainly fibrous, transparent or colorless. The microplastic composition of the upper soil layer was dominated by viscose, with a share of about 59%, followed by polyester and nylon, both with a share of 12%. PE had a share of 10%, while PET, PP and PE:PP had the same share of 2%. In the lower soil layer, viscose continued to be the main component of microplastics, accounting for 69%. Polyester and PET were next in line with 10% and 7%, respectively. In the lower soil layer, PP, PE, nylon and PE:PP microplastics were detected, all with the same share of 3% [88].

Microplastics undergo a slow degradation process in soil, mainly through biodegradation. This is conditioned by mechanical impact, oxidation processes and UV radiation. However, microplastics deposited in agricultural areas together with sewage sludge are difficult to decompose and persist for several years. The mass loss of microplastics in soil was only 0.1–0.4% after 800 days for PE and 0.4% after 1 year for PP [119].

In a study conducted in Scotland over 25 years of soil fertilization with sewage sludge, microfibers were found to be the dominant type of microplastic. Microfibers were detected in soils from all study plots and negative control plots in all years, accounting for 81–99% (average 92%) of the microplastics detected. This is consistent with the fact that the sewage sludge used in this study also contained predominantly microfibers (>65%) [116]. It can be stated that the high concentration of plastic fibers in agricultural soils indicates the use of sewage sludge for fertilization [116]. Weathering changes the structure of the microplastic crystal lattice, specific surface area and functional groups containing oxygen. This causes increased sorption of other pollutants. Studies have shown that microfibers and fragments are susceptible to degradation in terms of size changes. This creates the possibility of nanoparticle formation. On the other hand, microfilms (foils) are susceptible to degradation of surface features. Spherical microparticles are more resistant to degradation, but microfibers are also susceptible to loss of color [116].

#### 3.2.3. Microplastic Toxicity and Their Impact on Soil-Water Organisms

Microplastics present in soil pose a threat to plants and animals, affecting basic biochemical and morphological parameters, resulting in impaired growth, mass gain or, in the case of plants, the general condition of seedlings [120,121], and disruption of microbiological diversity and metabolic functions [122]. This is because microplastics easily accumulate in tissues. The greatest threat is posed by particles smaller than 100 μm, which can be absorbed by soil-dwelling organisms [121].

In order to investigate the impact of microplastics on plants, studies were conducted on lettuce, wheat and peas. For this purpose, microplastics with fluorescent markers were used, which facilitated the determination of the path of microplastic penetration and accumulation in plant tissues [121]. It was found that the largest fraction used was trapped and absorbed on the root surface due to the mucus secreted by the cap and did not penetrate through the root cortex or intercellular space to the apoplast. Scientists also examined the presence of nanoplastics in tissues and noticed that it penetrated through the roots, reaching the above-ground parts of plants and then penetrated many plant organs [121,123]. These studies proved that a significant part of microplastics is trapped in the rhizosphere of roots, which still constitute a source of microplastics for organisms feeding on them [121,124].

Microplastic fibers present in soil have been shown to have an inhibitory effect on plant root growth, a phenomenon that can be attributed to the reduction in nutrient and water availability they cause. Furthermore, these fibers have been shown to interact with root systems, resulting in mechanical damage [103]. In addition, the introduction of microplastics into soil may not only change the physicochemical properties of the soil and microbial populations, but also affect the enzymatic activity of the soil [83,125]. The physicochemical properties of microplastics, such as particle size and polymer density, affect the microbiological activity in the soil, thus influencing the metabolism of crops (changes in saccharides, amino acids and organic acids). As a result, the biomass of crops is reduced [126]. Omidoyin and Jho [127] observed significant changes in the rate of root colonization by arbuscular mycorrhizal fungi and concluded that the presence of microplastics may cause changes in microbiological functions [127].

Microplastics have a toxic effect on plants due to their ability to disrupt physiological processes, leading to oxidative stress and cell damage. Microplastics can penetrate plant structures, disrupt water and nutrient uptake, and affect the growth of roots, stems, and leaves. Their impact depends on their size, surface charge, and interactions with other contaminants, such as heavy metals [128]. Table 4 shows examples of the impact of microplastics on plants.

Microplastics, and especially nanoplastics, can cause serious physiological changes in plants, such as oxidative stress and damage to cell membranes, chloroplasts and mitochondria. As a consequence, apoptosis, or programmed death of plant cells, may occur. Plastic nanoparticles, which are characterized by a larger reactive surface, can penetrate plant tissues more effectively and thus cause greater damage, because they induce oxidative stress in plants. As a result, plants activate defense mechanisms, such as increased activity of antioxidant enzymes. Metabolism changes, main roots are shortened and the number of lateral roots increases. Nutrient uptake is also disrupted [128]. This may be related to the influence of microplastics on the production of chlorophyll a and chlorophyll b. The reduction in chlorophyll b production is associated with plant stress, which can be explained by the reduction in the pigment-protein in the photosynthetic process [82]. The phenomenon of increased production of one type of chlorophyll at the expense of the other type is also observed [134]. Iqbal et al. [135] determined the effect of PS and PE microparticles on maize yield, soil processes and consequently the enhancement of global warming potential. The addition of PS and PE microplastics with particle sizes of 75, 150 or 300 μm, reduced seed germination, plant height, aboveground plant biomass, grain yield and affected harvest efficiency compared to control samples without microplastics. The magnitude of changes in seed germination, plant height, and grain yield varied with particle size, whereas changes in aboveground plant biomass varied with polymer type and particle size. The strongest reduction in seed germination (−48%) was observed for particles of both polymers with a size of 75 μm. Microplastics of the smallest size caused the greatest reduction in plant growth, which were 30 cm shorter in the presence of PE [135]. The conditions that influence the uptake of microplastics by plants are still insufficiently studied, as is the adsorption of microplastics by plant roots. The impact of different types of microplastics, as well as nanoplastics, on vegetation, especially crops and edible plants, is worrying and poses a potential risk to public health.

Microplastics in soil relatively quickly secure sorption sites, forming unique colonies in the form of biofilms [136]. Studying the formation of biofilm on plastic surfaces, including microplastics, and its impact on the natural environment is another challenge for scientists. Determining how a specific biofilm affects the biochemical activity and colonies of microorganisms in soil used for agricultural purposes will allow for better management of the health care system [83].

According to Zhao et al. [137], the microbial colony structure in sewage sludge can be adjusted by qualitative changes in autotrophic and heterotrophic bacteria depending on the source and presence of microplastics. The decomposition of microplastics into nanoplastics is also important. It occurs in sewage sludge and leads to the formation of biofilms that produce reactive oxygen species. They are a key factor in oxidative stress reactions that have a severe inhibitory effect on other microbial colonies, including key enzymes, metabolic intermediates and end products [137].

Kwiatkowska and Ormaniec [83,95] investigated the ability of microplastics to form microbiological biofilms as a biospecific and active matrix attached to the cell surface. Polyethylene showed the greatest affinity for coliform bacteria. On the other hand, the same bacteria on polypropylene formed biofilms at a relatively slow rate, which could result in high concentrations for a longer period of time. In the case of mesophilic bacteria, no significant growth was observed on polyethylene and polyethylene terephthalate, it was within the moderate range. On the other hand, they show the lowest biofilm concentrations among polypropylenes. In the case of psychrophilic bacteria, polyethylene terephthalate, polyethylene and polypropylene have been found to have a relatively high affinity for biofilm formation. Differences in biofilm formation on the surfaces of different polymeric materials may result from chemical and physical interactions [95]. Microorganisms that decompose organic matter in the soil interact significantly with microplastics. Also, the complex network structure of microorganisms such as bacteria, fungi, protozoa and algae is an important component in agroecosystems. Any changes and inequalities in the functioning and structure of microbial colonies can affect the entire soil and plant system. Plants are linked to the soil microbiome and fauna. The microbial network affects growth and development, resistance to pathogens, productivity and yields, and the mobility and availability of nutrients [83]. In order to reduce the negative impact of microplastics on soil microorganisms, it is possible to use indole. Its action significantly increased the metabolic functions of soil microorganisms, including those related to predation or parasitism, the carbon cycle and the nitrogen cycle, while reducing the relative abundance of potential fungal pathogens. A beneficial regulatory effect of indole on the ecological functions of microorganisms in soil contaminated with microplastics can be observed [122].

Animal organisms can also be used as pollution indicators, so-called bioindicators. Bioindication consists of determining the state of the environment based on changes in the number, condition and general occurrence of a specific species. It is necessary to know its developmental requirements, its environmental adaptation and sensitivity to biotic and abiotic changes [121]. Among the species exposed to microplastics are earthworms, which obtain food by eating soil and the organic debris contained therein [121]. Earthworms of the species Eisenia andrei, Eisenia fetida, Lumbricus terrestris and Dendrobena veneta were used as bioindicators. All tested organisms showed the effect of microplastic pollution through increased concentration of oxidative stress markers and a slowdown in growth rate and the number of cocoons. Scientists [121] also showed that microplastics can enter tissues by using a fraction capable of fluorescence. Microplastics breached tissue barriers within the intestinal epithelium and longitudinal muscles and accumulated in muscles.

Earthworms are one of the most commonly used animal organisms in studies to determine the impact of microplastics in soil on animals living there. In some cases, it is not only the microplastics that are a problem, but also the substances that are transferred with them. As an example, a study by Sobhani et al. [138] showed that PVC has no acute effect on earthworm development, but at a concentration of 1000 mg PVC/kg, and in the presence of substances such as perfluorooctane sulfonate (PFOS) and perfluorooctanoic acid (PFOA), the reproductive activity of earthworms is reduced. PFOS and PFOA are transferred by microplastics [82].

Literature data [82] show that PE, mainly LDPE, less HDPE, has a decidedly negative effect on soil organisms. Huerta Lwanga et al. [63] studied the effect of litter containing microplastics (low-density polyethylene) for 2 weeks at concentrations of 0%, 7%, 28%, 45% and 60% on earthworms [139]. Due to the freezing of samples at the end of the study, the authors were unable to capture the effect of the microplastic dose on the weight loss of earthworms. However, this was achieved in previous studies [140] where it was concluded that LDPE concentrations >28% caused the greatest weight loss in *L. terrestris*. The results showed that concentrations of 28, 45 and 60% increased mortality and inhibited growth compared to the concentration of 7% [82,140]. The study of the effect of LDPE on *Eisenia fetida* showed that this microplastic causes damage to the nervous system and oxidative stress [141]. PE caused changes in the male reproductive organs of *Eisenia andrei*, which led to inhibition of spermatogenesis and reduced viability of earthworms [82]. Dead earthworms are food for *Lobella sokamensis* [142]. The authors studied the effect of microplastics in soil on the movement of *Lobella sokamensis* and showed that it has an inhibiting, limiting effect. Bazea et al. [143] determined the effect of LDPE, PS, PET, PP microplastics of 250 μm in size and concentrations of 2.5%, 5% and 7.5% (*w*/*w*) on *Lumbricus terrestris* and showed that microplastics were present in every segment of earthworms. It got there as a result of ingestion and resulted in the reduction in the surface mucus of earthworm membranes [82].

According to En-Nejmy et al. [82], PS is another microplastic that has significant effects on soil organisms. In the case of *Eisenia fetida*, Cao et al. [144] found that the effect of PS depends on its concentration. PS content at 1 and 2% (*w*/*w*) reduces the growth rate of *Eisenia fetida* and has a lethal effect [82].

Liu et al. [145] investigated the effect of microplastic concentration on soil nematode communities in the Qinghai–Tibet Plateau. Tests were conducted on prepared soil to which specific amounts of polyamide were added, i.e., 0%, 0.1%, 1.0%, 5.0%, and 10% (*w*/*w*). Nematode abundance, diversity, and metabolic traces were found to show a humpback response to microplastic treatments. That is, they reached a peak in samples with 0.1% polyamide content, after which lower values were achieved for subsequent samples. The presence of microplastics significantly affected the biomass of nematodes, with the lowest biomass observed in the 10% treatment. The presence of microplastics particularly affected the development of fungivorous and omnivorous nematodes, and this effect was stronger than in other trophic groups [145]. In the case of *Caenorhabditis elegans* nematodes, LDPE, PLA (polylactide) and PBAT (polybutylene adipate terephthalate) microplastics affected biodiversity by reducing the reproduction rate, understood as a decrease in the number of offspring compared to the control group of organisms. This effect was independent of the type of microplastics used [146].

Polyethylene terephthalate (PET) and polylactic acid (PLA) are preferred food of earthworms [147,148]. Literature data [82,149] indicate a negligible impact of PET microplastics on soil organisms. PET as a microplastic of fossil origin does not affect earthworms. In contrast to PLA, which induced an increase in the activity of glutathione peroxidase, the main function of which is to protect cells against oxidation by peroxides generated during biochemical processes, i.e., it was a response to the so-called oxidative stress. However, Song et al. [150] demonstrated a negative effect of PET on the digestive system of the snail Achatina fulica. After 28 days of exposure to PET microfibers, it was shown that changes occurred in the digestive organs, which influenced the way of feeding and excretion [82].

Another microplastic that affects the growth of soil organisms is PVC [151]. The impact of PVC is visible in the digestive system, as an imbalance in intestinal flora and consequently a reduction in growth rate. The presence of PVC microplastics has also contributed to a reduction in the rate of reproduction [82].

Despite numerous scientific publications, there are still many uncertainties regarding the impact of microplastics on terrestrial ecosystems. To assess their impact, it is necessary to take into account the type of polymer, the size of the particles and their concentration in the soil, their shape, the additives used and the degree of modification in the environment. Understanding the sources, characteristics and impact of microplastics on soils is crucial to develop effective strategies to reduce them and protect the integrity of terrestrial ecosystems, which is an important step towards sustainable environmental management.

### 3.3. Presence of Micro- and Nanoplastics in the Air

#### 3.3.1. Scale of Air Pollution by MNP

Micro- and nanoplastics in the air is an increasing challenge for both environmental protection and public health. Omnipresent around the world, plastics that enter the environment break down, contributing to the formation of microscopic particles MPs and NPs.

The resulting particles can travel through the atmosphere, affecting air quality and posing a potential threat to living organisms and [152]. Wind, precipitation, and other meteorological processes are considered the main pathways for the transport of microplastics, enabling the dispersion of micro- and nanoplastics over long distances. Jones et al. [153] stated, that one of the main research, one of the significant research challenges in this area is the difficulty of collecting air samples that are free from secondary microplastic contamination. This can lead to skewed results and limits the accuracy of analyses.

Le et al. [154] detected the presence of MNPs in both outdoor and indoor air at concentrations ranging from 0.0065 to 1583 (particles/m^3^). The identified sources include, among others, polyamide, polyester, polyethylene terephthalate, polypropylene, viscose, polyethylene, polystyrene, polyvinyl chloride, polyacrylonitrile, and ethylene-vinyl acetate.

#### 3.3.2. Sources of MNP in the Air

It is estimated that an individual inhales approximately 15 m^3^ of air daily, with an average exposure time to air of around 10 h, making air one of the main sources of human exposure to MNPs. Particularly hazardous are microplastic fibers of micron size or smaller due to their high prevalence and strong atmospheric transport potential [155,156,157].

According to Cox et al. [158], annual human intake of microplastics may range from 74,000 to 121,000 particles, with about half of this exposure occurring through inhalation. Cataro et al. [159] provided an interesting comparison of microplastic exposure from ingestion. They tested contaminated mussels and compared them to home-cooked meals that had been exposed to contamination from fiber fallout. They concluded that fibers pose a greater risk to humans.

Additionally, epidemiological data collected in the United States, Canada, and the Netherlands have recently shown an increased incidence of respiratory diseases, such as coughing, shortness of breath, occupational asthma, and interstitial lung disease, among workers handling nylon, polyester, and polyamide fibers, suggesting the harmful effects of inhaling high doses of airborne microplastics.

The occurrence, transport range, and deposition of micro- and nanoplastics in the atmosphere remain active areas of research. The atmosphere is considered an especially efficient carrier of anthropogenic pollutants, including semi-volatile substances and particulate matter [160,161]. While the transport of particles such as black carbon, tire debris, or nanoparticles has been extensively studied, the transport of micro- and nanoplastics particularly over oceans still involves high uncertainty and methodological challenges.

Airborne microplastics originating from tire wear can account for up to 84% of the plastic present in the air. These particles are released through the abrasion of tire treads or thermal degradation of tires, creating both fragmentary and fibrous particles of various sizes [162,163,164,165].

Another recognized source of airborne microplastics is the migration of particles from water to air via bursting bubbles. Kjærgaard et al. [166] and Zha et al. [167] explained the transfer of polystyrene particles via bursting bubbles in low-salinity water under laboratory conditions.

Meanwhile, Zheng et al. [168] investigated microplastics in atmospheric deposition. The concentration of submicron polystyrene particles in the air increases after air masses pass through waste incineration plants, further suggesting that such facilities could be a source of these particles [169].

Mingkai Xu et al. [170] highlighted the growing number of reports documenting microplastics, including polystyrene (PS), in air samples, pointing to a potential threat to the human respiratory system. Furthermore, studies by Stapleton [171] indicate that factors such as exposure time, particle diameter, and concentration are crucial in assessing the toxicity of PS nanoplastics to alveolar epithelial cells.

Inhaled nanoplastics suspended in the air may pose risks; however, the understanding of their toxicity and the mechanisms of their action are still largely unknown. Improving our knowledge of the transport of nanoplastics in the air allows us to obtain new information on their toxicity in the human respiratory system [172,173].

#### 3.3.3. The Impact of Airborne Microplastics on Human Health

Microplastics and nanoplastics have been discovered and studied in nearly all environmental compartments; however, the current body of data regarding their role as sorbents and carriers of other hydrophobic compounds in the atmosphere remains limited. Existing findings suggest that the carrier function of PP (polystyrene) and PE (polyethylene) for selected micropollutants is due to monotonic correlations between these polymers and 23 selected polycyclic aromatic hydrocarbons (PAHs). High correlations were observed primarily for low-molecular-weight congeners and were more pronounced than those recorded between PAHs and other PM2.5 components, such as elemental carbon and organic matter again indicating unique interactions between these emerging pollutants [174].

Eberhard et al. [175] analyzed inhalation exposure to microplastics (MPs), demonstrating significant differences across age groups. The highest levels of absorbed MPs were observed in infants, followed by preschool children, high school students, pregnant women, adolescents, and non-pregnant adults. The doses of MPs used in toxicology studies resulted in higher mean inhalation exposure levels than those detected in environmental samples.

Falsini also reported the negative impact of plastic air pollution on plants [176]. Research by Lin et al. [177] indicates that particle size is crucial for nanoplastic internalization and contributes to mitochondrial damage in human respiratory cells, posing a threat to health. Nonetheless, uncertainties remain regarding the toxicological effects and health risks associated with low-level, everyday inhalation exposure to microplastics.

MNPs have been detected in air, drinking water, food, and even inside living organisms, raising concerns about their potential impact on reproductive and developmental health. To address this, MNP content has been studied in various human reproductive tissues, including semen, placenta, and ovarian follicular fluid, as well as in reproductive tissues of various animal species. Studies have documented the presence of MNP in these organs, suggesting a potential association between MNP exposure and an increased incidence of infertility and adverse pregnancy outcomes [178]. This is based on the evidence that MNPs negatively affect reproductive parameters, including sperm quality, ovarian function, and steroidogenesis. In the male reproductive system, MNPs disrupt testicular tissue structure, impair reproductive endocrinology, and reduce sperm quality. In females, MNPs affect the structure and function of ovarian tissue, interfere with hormone secretion, and influence the endometrium and embryo implantation. Moreover, MNPs cause developmental toxicity in animal models, affecting embryonic development and offspring health, and can induce transgenerational effects. Notably, an in depth literature review suggests a key role of oxidative stress, inflammation, and epigenetic modifications in MNP induced toxicity [178].

Concerns about the toxic health effects of micro- and nanoplastics are growing, but research is still limited. Animal and laboratory studies may to some extent indicate the dangers these particles pose to human health. Although studies on mammals are highly valuable, only a few reports are available. For instance, animal studies have shown that the absorption of plastics through inflamed mucosal areas depends on particle size especially for smaller plastic particles which can interact bimolecularly [155]. Unlike traditional air quality indicators such as PM2.5 and PM10, plastics and their progressive degradation contribute to global microplastic air pollution, with no clear distinction between developed and developing countries.

Elevated atmospheric microplastic concentrations have been recorded in London (UK), Paris (France), Tehran (Iran), Shanghai (China), and Hamburg (Germany) [179], indicating a significant public health threat and global regulatory challenges.

Therefore, studies should be conducted to identify the health risks and molecular mechanisms of action of inhaled microplastics, using both classical and innovative toxicological models.

The atmosphere plays a key role in the global long-range transport of micro- and nanoplastic particles [178,180]. Few studies exist on the scale, variability, and uncertainty of human exposure to airborne micro- and nanoplastics [158,159,181], and much remains unknown about the toxicological effects and health risks associated with low level inhalation exposure to microplastics [179,182,183].

## 4. The Effects of Test Particles on Human Health

Scientific reports confirm the presence of MPs in various organs of the human body and analysis related to their impact on health is a very important research issue.

An important piece of information in the process of health risk assessment is data that allows for the estimation of the mass of plastic entering the human body. In studies based on an analysis of data from fifty-nine publications, researchers calculated the average mass of individual microplastics within the size range of 0–1 mm. They estimated that, on average, approximately 0.1–5 g of microplastic enter the human body through various routes per week [184].

Research suggests that the efficiency of microplastic (MP) particle capture by the epithelium of the respiratory or digestive tract likely increases as particle size decreases [185]. Smaller particles are more readily absorbed by the digestive system than larger ones [186]. Researchers also indicate that these smaller MPs and nanoplastics (NPs) are generally considered more hazardous to human health [187,188]. As particle size diminishes, the probability of systemic dissemination rises markedly. Ultrafine fragments measuring 10 µm or less can readily cross cellular membranes, enter the circulation and subsequently reach sensitive organs—including the liver, spleen and even neural tissue. [189,190]. The presence of microparticles in the air presents a pathway for their entry into the respiratory system [191,192].

Microplastics can affect human health both directly and indirectly [187,193]. The indirect impact results from the fact that their surfaces often provide a favorable environment for the growth of pathogenic microorganisms and simultaneously serve as carriers for the development and transport of pathogens. Through this mechanism, chemical pollutants, antibiotics, organic contaminants, and pesticides can also be transported and deposited [194]. Microplastics on synthetic materials promote the formation of biofilm, although it differs from that formed on natural substrates. This biofilm acts as a reservoir for microorganisms, and studies suggest it may also facilitate the horizontal gene transfer between microbes, which may include the transfer of antibiotic resistance genes [195].

The direct impact, as shown in studies, depends on the routes and sources of exposure. Most commonly, the gastrointestinal tract is identified as the primary route through which MNPs enter the body. Multiple investigations have detected micro- and nanoplastic (MNP) contamination across a broad spectrum of foodstuffs and beverages—ranging from fish and crustaceans to drinking water, tea, beer, wine, energy and soft drinks, milk, table salt, sugar, honey, poultry products, fruits and vegetables [196,197,198,199]. MNPs have likewise been isolated from diverse human biological matrices, including blood, urine, feces, sputum, breast milk, hair and saliva [188,189,200,201,202,203,204]. The detection of MPs in human blood is particularly significant, as their presence in the bloodstream poses a risk of transport to various organs [205,206]. In addition to the circulatory system, they are also capable of penetrating the lymphatic system [189,203,207,208,209].

Once lodged within the human body, plastic microparticles can provoke site-specific effects—including inflammatory reactions, oxidative stress, apoptotic pathways, immune modulation, mechanical injury and metabolic dysregulation at the cellular level [193,210,211,212,213].

Research has also been conducted on carotid arteries to analyze the presence of MNPs in atherosclerotic plaques, aiming to determine whether their presence could be a potential cardiovascular risk factor. The study found that patients in whom MNPs were detected in carotid artery plaques had a higher risk of heart attack, stroke, or death compared to those in whom MNPs were not found in the plaques [214].

Studies have confirmed the impact of micro- and nanoplastics (MNPs) on the development and exacerbation of autoimmune diseases characterized by chronic inflammation, such as arthritis and inflammatory bowel diseases (IBD). Within the group of joint inflammatory diseases involving inflammation of the synovial membrane, rheumatoid arthritis and joint symptoms of systemic lupus erythematosus were analyzed. Inflammatory bowel diseases (IBD), involving inflammation of the gastrointestinal tract wall, were studied in the context of Crohn’s disease and ulcerative colitis [215,216,217]. The ingestion of MNPs disrupts immune homeostasis in both the gastrointestinal tract and joints. It leads to the aggravation of joint inflammation by influencing, among other things, the proliferation and migration of synovial membrane cells [218]. It has also been shown that MNP particles present in the intestines reduce the production of mucin, the mucus that serves as a protective barrier for intestinal epithelial cells [219].

Ultrafine plastic particles have been detected in neural tissue and shown to traverse or accumulate within the placenta, as documented in both vaginal and cesarean deliveries [212,220,221,222]. Pregnant individuals are therefore particularly susceptible to associated endocrine-disrupting chemicals, which can cross the placenta, lodge in developing brain tissue and potentially impair fetal neurodevelopment [223,224].

Exposure to MNP particles may impact male fertility by significantly affecting sperm quality, thereby reducing the likelihood of conception [189,225]. The consumption of microplastics has been strongly associated with diseases such as infertility, obesity, cancer, and others [226,227,228].

MNPs may cause both acute and chronic toxicity, including neurotoxicity, immunotoxicity, genotoxicity, and cytotoxicity [229]. A report on MNPs states that toxic compounds can have long-term effects on human health, including disruption of the endocrine system, induction of mutagenicity, and carcinogenicity [230]. These substances may interfere with the synthesis, secretion, transport, and elimination of natural hormones essential for maintaining homeostasis [224,231], cause changes in fat metabolism, and contribute to health problems such as diabetes and obesity [232].

## 5. Regulating Nano- and Microplastics

Growing awareness of the dangers of micro- and nanoplastics (MNPs) in the environment has led to a number of global initiatives and regulations aimed at reducing their negative impact. A global example of action in this regard is the establishment of Agenda 2030 by the United Nations (UN), which supports taking action to combat the MNP pro-blem [233]. UNEA Resolution 5/14 established an Intergovernmental Negotiating Committee (INC) to develop a binding anti-plastic pollution treaty covering the entire life cycle of plastics [234].

In 2022, the United States and the European Commission formally joined the initiative, pledging their support for efforts to reduce plastic waste in the oceans. The Organisation for Economic Co-operation and Development (OECD) has issued recommendations regarding unintentional microplastic emissions from tires and textiles, offering member states ready-made solutions in this area [235].

In 2018, the European Commission adopted a plastics strategy aimed at protecting the environment, reducing marine litter, and improving the design and recycling of products within the European Union [236]. In 2024, Delegated Directive 2024/1441 was issued, supplementing Directive 2020/2184, introducing a methodology for measuring microplastics in drinking water [237].

Regulation (EC) No 1907/2006—REACH (Registration, Evaluation, Authorisation and Restriction of Chemicals) is a fundamental legal act of the European Union concerning the control of the safety of chemical substances placed on the market [238]. With regard to microplastics, it also covers chemical substances that may be components of microplastics and may affect human health. Under REACH provisions, all substances must be assessed for safety, including their presence in everyday products such as cosmetics and cleaning agents, which may contain microplastics.

Commission Regulation (EU) 2023/2055 of 25 September 2023 is one of the key EU legal acts introducing a gradual ban on the use of microplastics in consumer products such as cosmetics and detergents [239]. This process began on 16 October 2023 with the prohibition of the sale of cosmetics containing plastic microbeads (e.g., exfoliants, loose glitter). Subsequent stages include a ban on the use of microplastics in rinse-off cosmetics, such as shampoos and shower gels (from 17 October 2027), followed by a ban on their use in leave-on cosmetics, such as creams and lotions (from 17 October 2029). Finally, as of 17 October 2035, the use of microplastics in make-up and nail styling products will be prohibited.

Directive (EU) 2019/904 of 5 June 2019—adopted by the European Parliament and the Council—focuses on curbing single-use plastics and items that contain microplastics, thereby preventing their breakdown into micro- and nanoplastic particles [240]. While the main objective of the directive is environmental protection, its provisions are also relevant to human health, as micro- and nanoplastics present in the air, water, and food may pose a risk to the human body.

Directive 2008/98/EC on waste establishes the principles of waste management, including plastic waste that may degrade into MNP (micro- and nanoplastics) in the environment [241]. Although it does not directly address public health, reducing environmental pollution contributes to the protection of human health, particularly by decreasing exposure to MNP present in water and food.

Regulation (EU) 2020/2081 introduces restrictions on the use of microplastics in cosmetics and other everyday products, such as exfoliants, shampoos, and shower gels. Its aim is to minimize health risks associated with the penetration of microplastics into the human body through the skin, digestive system, or respiratory tract [242].

The European Union is implementing a range of regulations aimed at monitoring and controlling environmental pollution, including microplastics, which may affect human health. Table 5 presents a summary of legal regulations concerning the environmental impact of microplastics.

Some countries have introduced regulations limiting the use of microplastics in industry, with most of them focusing on cosmetic and personal care products, which are the main source of primary microplastics [248,249,250].

California [251] and the United Kingdom [252] were pioneers in efforts to reduce the use of plastic bags by introducing bans or fees for their use, which also serve as a source of revenue for legislators [253]. Complete bans have also been implemented in countries in Africa (Cameroon [254], Morocco [255], Kenya [256]), Asia (China [257], Taiwan [258], Malaysia [259]), and Europe (France [260], Italy [261]).

In developing countries such as Kenya, these regulations represent an important step in combating plastic pollution, although the effectiveness of enforcement remains inconsistent [262,263,264,265].

In summary, despite global efforts to reduce micro- and nanoplastic pollution, the effectiveness of these actions varies by region. The European Union is implementing regulations, but their impact is limited by enforcement challenges. Developing countries face a lack of infrastructure, while major economies such as the United States and China still lack comprehensive legislation on microplastics.

## 6. Conclusions and Directions for Further Research

MP and NP pose a significant threat to human health. Studies have demonstrated its occurrence in food, its presence in the respiratory and digestive systems, and its occurrence in the bloodstream, which allows it to enter many organs via the blood route. The results of the study indicate an association with the risk of oxidative stress, changes in cell division and viability, DNA damage, immunological reactions, and increased risk of cancer, respiratory and neurodegenerative diseases, and inflammatory conditions.

Numerous studies show that micro- and nanoplastics present in fresh and salt water negatively affect various aquatic organisms. Fresh water as well as aquatic organisms containing micro- and nanoplastics consumed by humans pose a real threat to human health [266,267].

Microplastic pollution is a major environmental problem. In the case of marine and ocean waters, data are available on MP concentrations and composition, as well as their effects on aquatic organisms. For example, studies of microplastic content in the Southern Ocean have shown the presence of about 100 molecules-m^−2^ [268], in the Mediterranean Sea near Corsica about 0.2 molecules-m^−2^, in the Pacific (off the coast of southern California) about 8 molecules-m^−3^ [269], and in the East China Sea < 0.2 molecules-m^−3^ [270]. Contamination of the marine environment with microplastics occurs, among other things, through runoff from river waters. On the other hand, studies on the pollution of fresh surface waters with MNP are conducted much less frequently than is the case with seas and oceans. In Poland, this problem is practically unrecognized, and foreign data indicate that the number of microplastic particles in river waters is highest in estuary sections and urbanized areas, and depends, among other things, on atmospheric conditions [271,272]. Standing waters, i.e., lakes, dam reservoirs, are also exposed to MNP pollution.

Based on literature sources, it has been shown that any type of microplastics found in sewage sludge poses a threat to soil and plants, confirming their negative impact on soil ecosystems. Therefore, it is important to note that long-term use of such sludge in agriculture can lead to permanent changes in the soil, thereby reducing its ability to produce plants and support biological life. Microplastics are also present in compost, which is also used to fertilize the soil. In light of the above data, it is necessary to characterize microplastics in order to determine their impact on the soil ecosystem. It should be noted that potential impacts on soil ecosystem functioning may be caused by microplastics, as well as secondary micro- and nanoplastics and dyes from microfibers. Once released, microplastic particles can migrate beyond their initial deposition site, expanding their ecological footprint. Moreover, they can transport co-occurring contaminants found in sewage sludge—such as organic xenobiotics, pathogenic microorganisms and heavy metals—thereby intensifying disruptions to soil functioning and elevating risks to animal and human health. Accordingly, a rigorous appraisal of microplastic-related environmental effects in terrestrial ecosystems—and the formulation of effective management interventions—demands the systematic integration of these variables [273,274].

Greater research emphasis is warranted on the presence of microplastics in agricultural soils, as these particles can enter trophic networks, jeopardizing human health and food security. Priority should be given to elucidating their effects on soil fauna, crop physiology and the wider soil biota. Of particular interest are some of the non-toxic effects. Scientists want to learn more about how these molecules affect the pace of ecosystem processes and how they interact with other factors of global change.

Microplastics and nanoplastics have been identified in almost all environmental ecosystems, but the body of data on their contribution and role as a sink and no-see-um for other hydrophobic compounds in the atmosphere is limited. It has been demonstrated that micro- and nanoplastic particles can be transported in the atmosphere by phenomena such as wind, rain, snow, and fog. This allows them to spread on a large scale, both in cities and rural areas, and even in remote places such as mountains and oceans. However, there is still a lack of extensive and detailed studies to determine the amount of MNP particles in the air and regulations to determine the testing methodology and standards for testing MNPs in the air.

Microplastic and nanoplastic pollution is becoming increasingly prevalent in aquatic, soil, and air environments. The European Union is introducing directives that require member states to incorporate microplastic testing regulations into national laws, but implementation takes time. Developing countries face challenges due to limited testing infrastructure, and major economies such as the United States and China still lack comprehensive standards and legislation on microplastics. Effectively addressing this issue requires global cooperation and the standardization of testing methodologies.

## Figures and Tables

**Table 1:** Examples of polymers and their sources.

Type of Polymer	Abbreviation	Polymer Source
Polyamide	PA	Synthetic fibers from clothing (e.g., nylon)
Polyester	PES	Sports and casual clothing, home textiles [16,17]
Polyethylene terephthalate	PET	Disposable bottles and packaging, plastic, textiles [18]
Polypropylene	PP	Food packaging, disposable utensils, filters, fibers in masks and hygiene materials [19,20]
Viscose	CV	Regenerated fibers from cellulose used in textiles and wipes [21]
Polyethylene	PE	Plastic bags, packaging films, bottles, microbeads from cosmetics and detergents [20,22]
Polystyrene	PS	Disposable packaging, polystyrene, building insulation, foam material fragmentation [22]
Polyvinyl chloride	PVC	Pipes, liners, building components [23]
Polyacrylonitrile	PAN	Synthetic fabrics (e.g., fleece), ropes, filters
Ethylene vinyl acetate	EVA	Shoe soles, mats, toys, sports materials [24]

**Table 2:** Examples of polymers in aqueous environments and their sizes.

Type of Polymer	Abbreviation	Size of Polymer [Ref.]	Concentration [Ref.]
Polyethylene terephthalate	PET	350 μm [28,29], >100 μm, ≥75 μm, 65.2 ± 41.9 μm, ≤49 μm, 15 μm, 10–40 μm [40], 5 μm [42]	0.41, 9.9 ± 2.5, 175 MP·m^−3^ [42], 0.8–4.000 MP·ml^−1^ [39,40]
Polyamide	PA	180.5–118.7 μm [31], <50 μm, 22.7 μm	0–219.6, 0.02–2.38 MP·m^−3^ [42,45]
Polyester	PES	350 μm [28,29], ≥75 μm [42], 4–7 μm [42]	2.19 MP·m^−3^ [42]
Polypropylene	PP	380 μm, 200 ± 90 μm, 50 ± 26 μm [29], ≥15 μm, 85 μm, 5 μm <10 μm [41,42]	10,000 MP·dm^−3^, 680 MP·dm^−3^ [29], 9.9 ± 2.5 MP·m^−3^ [42]
Polyethylene	PE	≥15 μm, <10 μm [42,43]	9.9 ± 2.5 MP·m−3 [42]
Polystyrene	PS	100–400 μm [28,29], 180.5–118.7 μm [31], <10 μm, 5 μm [42]	9.9 ± 2.5 MP·m^−3^ [42]
Polyvinyl chloride	PVC	<10 μm, 5 μm [42]	9.9 ± 2.5 MP·m^−3^ [42]
Polymethyl methacrylate	PMMA	<10 μm, 5 μm [42]	9.9 ± 2.5 MP·m^−3^ [42]

**Table 3:** EI values for sample vegetation.

Vegetation	Location	EI	Ref.
Sea grass *H. ovalis*	South China Sea coast	1.3	[109]
Mangrove forest *A. marina*	South China Sea coast	17.6	[109]
Sea grass *E. acoroides*	Hainan in China	2.1; 2.9	[109]
Meadow *Zostera marina*	Scotland	1.7	[109]
Inland and riverside vegetation	Italy, the riverine area of the Tiber, Aniene, Almone, Mignone, Marta, Sacco, Ninfa-Sisto, Arrone rivers	17.6	[110]

**Table 4:** The impact of microplastics on plants.

Plant	Microplastic		Ref.
	Type	Impact	
Soybean (*Glycine max* L.)	PS, PE, PVC (C = 10%)	Increased plant biomass and reduced root to shoot ratio.	[126]
Onion (*Allium cepa* L.)	PS (50 nm)	Decreased mitotic index, genotoxicity (cytogenetic anomalies and micronucleus formation), and increased oxidative stress.	[129]
Rice (*Oryza sativa*)	PS (100 nm and 1 μm) at 0, 0.1, 1 and 10 mg L^−1^	Reduction in primary root length, inhibition of nutrient uptake, stimulation of lateral root growth to meet nutrient requirements.	[130]
Lettuce (*Lactuca sativa* L.)	5 type	Inhibition of plant growth, root lignification, root cell apoptosis and oxidative stress.	[128]
Lettuce (*Lactuca sativa* L.)	PE (40 μm; 0.1 and 1% *w*/*w*)	Decrease in the shoot fresh weight, shoot dry weight, leaf number, Chlorophile a, leaf N, P, K content.	[131]
Strawberry plants (*Fragaria × ananasa Duch*)	HDPE 2–5 mm 0.2 g kg^−1^	Decrease in plant height, biomass, stem diameter, and root surface area, volume and biomass of root.	[132]
Mustard (*Brassica juncea var. Multiceps*)	PE (6.5 ± 1.9 nm; 1% *w*/*w*) PP (7.8 ± 1.6 nm; 1% *w*/*w*)	Increase in plant height and early senescence and flowering with small leaves and reduction in root length and Chlorophyll b content.	[133]

**Table 5:** Regulation of microplastics in the environment.

Environment Area	Main Acts	Range of Adjustment
Water	Water Framework Directive (2000/60/WE) [243] Regulation (UE) 2020/741 [244] Directive 2019/883 [245]	Protecting surface, ground and drinking water Monitoring the presence of microplastics Reducing microplastic emissions from land-based sources
Soil	Directive 2008/98/EC on waste [241] Regulation REACH (WE 1907/2006) [238] Directive 2019/904 [240]	Managing plastic waste Reducing the release of microplastics into the soil Bans the use of certain single-use plastic
Air	Directive 2008/50/WE [246] Regulation REACH (WE 1907/2006) [238] Directive 2013/39/UE [247]	Monitoring of particulate matter (which may contain MP) Reduction in chemical emissions into the atmosphere

## Data Availability

Not applicable.

## Abbreviations

The following abbreviations are used in this manuscript:

ACR	Acrylic polymer/poliakrylan
BPA	Bisphenol A
BPS	Bisphenol S
CV	Viscose
DOC	Dissolved Organic Carbon
EC	Environmental Index
EI	European Commission
EVA	Ethylene-Vinyl Acetate
EU	European Union
HDPE	High-Density Polyethylene
INC	Inter-governmental Negotiating Committee
LDPE	Low-Density Polyethylene
MP	Microplastics
MNP	Micro- and Nanoplastics
NP	Nanoplastics
MPC	Microplastic Carbon concentration
OECD	Organisation for Economic Co-operation and Development
PA	Polyamide
PAHs	Polycyclic Aromatic Hydrocarbons
PAN	Polyacrylonitrile
PBAT	Poly(butylene adipate-co-terephthalate)
PBBs	Polybrominated Biphenyls
PES	Polyester
PET	Polyethylene Terephthalate
PFOS	Perfluorooctane Sulfonate
PFOA	Perfluorooctanoic Acid
PHB	Poly(3-hydroxybutyrate)
PLA	Polylactic Acid
PP	Polystyrene
PVC	Polyvinyl Chloride
REACH	Registration, Evaluation, Authorisation and Restriction of Chemicals
UV	Ultraviolet
UN	United Nations
UNEA	United Nations Environment Assembly
